# Adverse Event Reporting Patterns Associated with Concomitant Use of 5-HT3 and NK-1 Receptor Antagonists: A Large-Scale Spontaneous-Reporting Analysis

**DOI:** 10.3390/ph19101626

**Published:** 2026-10-14

**Authors:** Ruiqi Zhao, Shiqi Tang, Mengyao Han, Hui Hu, Jiesheng Guo, Minling Lv, Jialing Sun, Xiaozhou Zhou

**Affiliations:** 1 The Fourth Clinical Medical College, Guangzhou University of Chinese Medicine, Shenzhen 518033, China; 20252120304@stu.gzucm.edu.cn (R.Z.);; 2 The Second Clinical Medical College, Guangzhou University of Chinese Medicine, Guangzhou 510006, China; 3 Faculty of Chinese Medicine, Macau University of Science and Technology, Taipa, Macao 999078, China

**Keywords:** pharmacovigilance, CINV, adverse drug events, FAERS, 5-HT3 RAs, NK-1 RAs

## Abstract

**Background/Objectives**: Chemotherapy-induced nausea and vomiting (CINV) is common and compromises quality of life and adherence. 5-HT3 receptor antagonists (RAs) and NK-1 RAs are cornerstones, yet adverse event patterns associated with their concomitant use are not well characterized. **Methods**: We queried the US Food and Drug Administration Adverse Event Reporting System (FAERS) from 2004 Q1–2025 Q1 and deduplicated reports per Food and Drug Administration (FDA) guidance. Five algorithms—the Ω shrinkage measure, additive model, multiplicative model, combination risk ratio, and chi-square model—detected disproportionate reporting signals associated with concomitant use. We also performed time-to-onset (TTO) analysis and logistic regression. **Results**: We identified 965 concurrent use cases with a mean age of 58 years and a mean weight of 70 kg, with 59.7% (*n* = 575) of these cases being female. These reports included 366 preferred terms (PTs). Forty-nine PTs were significant across all five algorithms, mainly skin and subcutaneous disorders, hypersensitivity reactions, blood pressure abnormalities, hepatic and renal impairment, and nervous system disorders, with additional signals for abnormal antidiuretic hormone secretion, respiratory disorders, and oropharyngeal disorders. TTO showed most adverse drug events (ADEs) occurred within 20 days of therapy initiation. Logistic regression indicated that older age was associated with higher odds of classification as an important medical event (IME). **Conclusions**: Beyond prescribing information, we detected previously unrecognized adverse event terms that were disproportionately reported with concomitant use and that merit attention in practice.

## 1. Introduction

According to Global Cancer Statistics 2022, there were nearly 20 million new cases and approximately 9.7 million deaths worldwide; cancer may surpass cardiovascular disease as the leading cause of premature mortality in most countries this century [1,2]. Despite ongoing advances in oncologic therapeutics, chemotherapy remains the cornerstone of cancer treatment. Chemotherapy-induced nausea and vomiting (CINV) is a common and serious complication; suboptimal control can precipitate treatment delays, dose reductions, emergency interventions, increased healthcare utilization, and even premature discontinuation of chemotherapy, thereby severely compromising patients’ quality of life and undermining adherence [3]. Multinational Association of Supportive Care in Cancer (MASCC), European Society of Medical Oncology (ESMO), American Society of Clinical Oncology (ASCO), National Comprehensive Cancer Network (NCCN) among others, have successively published evidence-based antiemetic guidelines [4,5]. With the increasingly widespread clinical use of 5-Hydroxytryptamine Type 3 Receptor Antagonists (5-HT3 RAs) and Neurokinin-1 Receptor Antagonists (NK-1 RAs), triple- and quadruple-agent antiemetic prophylaxis regimens have demonstrated favorable efficacy in managing moderately emetogenic chemotherapy (MEC) and highly emetogenic chemotherapy (HEC).

In recent years, prophylactic antiemetic therapy has advanced substantially, and combination regimens incorporating 5-HT3 RAs and NK-1 RAs have gained broad acceptance. However, even if these combinations have clinical benefits, we must be concerned about the risk of associated ADEs, including arrhythmias, hypersensitivity and infusion reactions, hepatic enzyme elevations, blood-pressure abnormalities, neurologic symptoms, and potential pharmacokinetic interactions driven by CYP3A4 modulation by NK-1 RAs. Prior investigations have evaluated ADEs associated with 5-HT3 RAs or NK-1 RAs [6,7], but typically examined each class in isolation. Individual agents within each class differ in their metabolism and interaction potential; however, the present analysis was conducted at the class level because FAERS records frequently do not reliably distinguish among individual agents in combination reports. ADEs with their concomitant use have been only cursorily addressed in meta-analyses [8,9], and the potential safety concerns associated with their combined use remain uncertain. Recent evidence indicates that, across MEC and HEC, patients receiving combined 5-HT3 RAs and NK-1 RAs are more likely to experience severe ADEs [10].

In summary, leveraging Food and Drug Administration Adverse Event Reporting System (FAERS)—the world’s largest pharmacovigilance database of drug-related adverse events—we conducted a pharmacovigilance assessment of adverse event reporting patterns associated with concomitant 5-HT3 RA and NK-1 RA use, with particular attention to underreported and high-priority ADEs. This study leverages FAERS to identify and characterize safety signals disproportionately reported with concomitant 5-HT3 RA and NK-1 RA use, delineate time-to-onset patterns, and highlight patient-level characteristics associated with IME classification to inform pharmacovigilance and targeted clinical monitoring.

## 2. Results

### 2.1. Descriptive Characteristics

**Figure 1:**
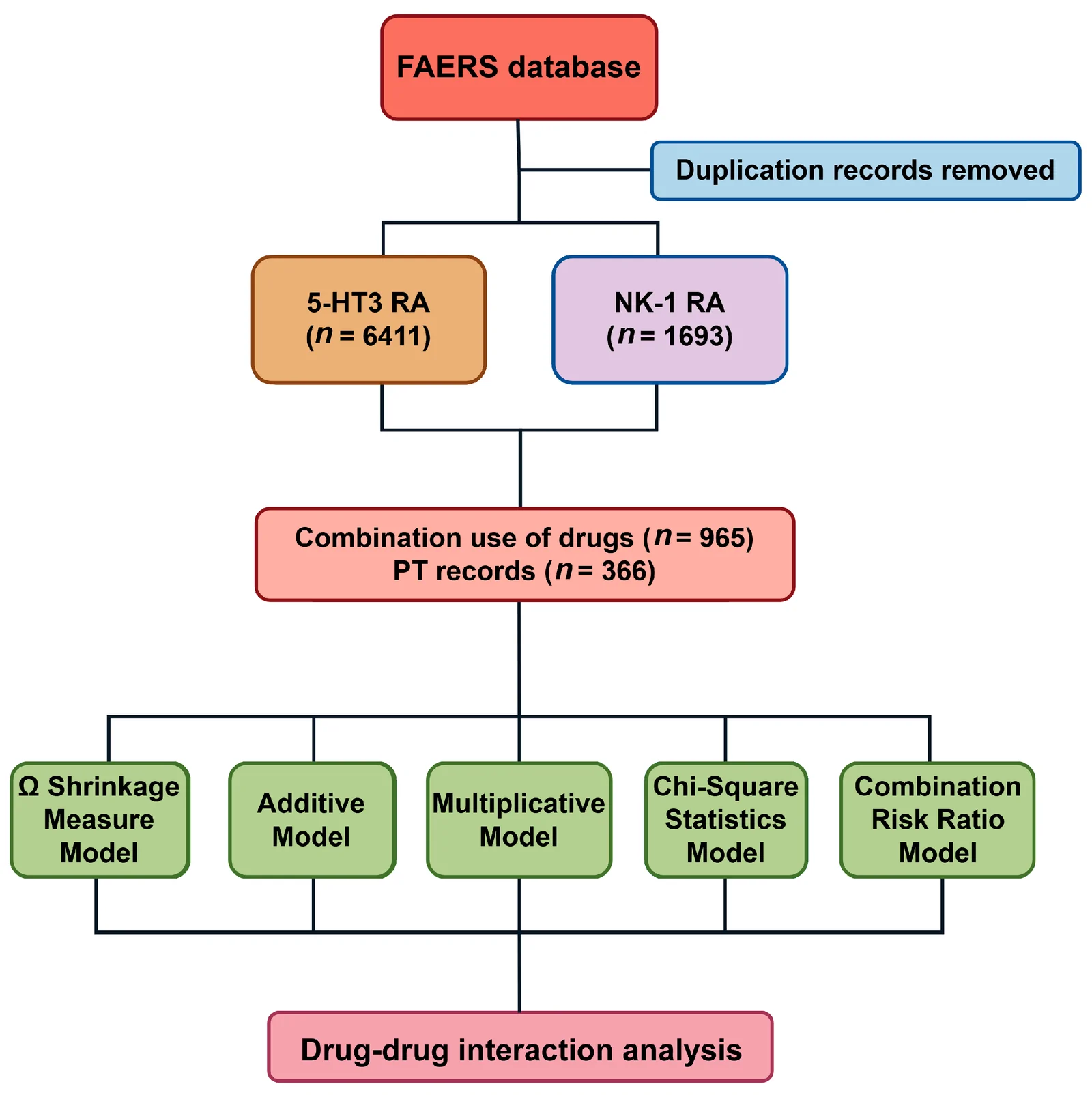
Detailed technical roadmap of this study. FAERS, FDA Adverse Event Reporting System; 5-HT3 RA, 5-Hydroxytryptamine Type 3 Receptor Antagonist; NK-1 RA, Neurokinin-1 Receptor Antagonist; PT, preferred term.

We retrieved 21 years of FAERS data spanning Q1 2004 through Q1 2025; the detailed ADE report-screening workflow is shown in Figure 1. In total, we identified 6411 reports listing 5-HT3 RAs as the PS, 1693 reports involving NK-1 RAs, and 965 reports describing concomitant use of both classes.

**Figure 2:**
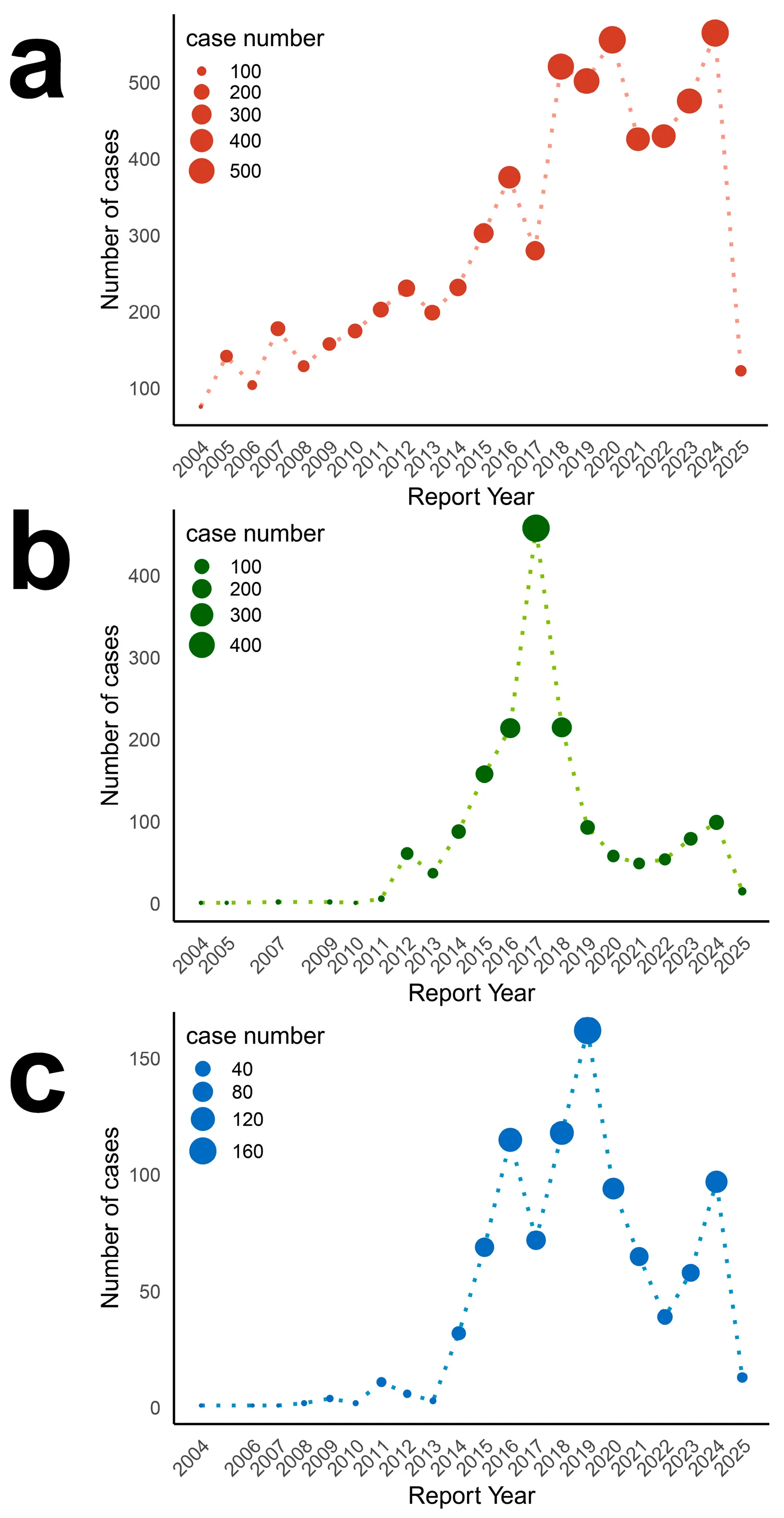
The number of reports of adverse drug events each year from the first quarter of 2004 to the first quarter of 2025. (**a**) 5-HT3 RA; (**b**) NK-1 RA; (**c**) Combined Use of 5-HT3 RA and NK-1 RA.

Viewed by year, ADE report counts associated with 5-HT3 RAs and NK-1 RAs have increased only modestly since market introduction. For 5-HT3 RAs, counts began to rise in 2006, peaked around 2020, and have continued to trend upward in recent years (Figure 2a). By contrast, NK-1 RAs-related ADEs surged beginning in 2012, peaked in 2017, declined rapidly, and rose again in 2021 (Figure 2b). Notably, ADEs linked to concomitant 5-HT3 RA and NK-1 RA use increased from 2013, reached their highest level in 2019, and showed a renewed upswing in 2022, with considerable year-to-year volatility (Figure 2c). Only Q1 2025 data were included, yielding relatively few reports; changes in raw report counts may reflect drug utilization, market availability, and reporting practices rather than changes in adverse-event frequency, and ongoing surveillance remains warranted.

Demographic characteristics (Table 1) were as follows: mean age, 58 years; mean body weight, 70 kg; women, 59.7% (*n* = 575); men, 40.3% (*n* = 388). By report source, 92.3% (*n* = 876) were submitted by healthcare professionals (MD, HP, PH, OT); geographically, 55.5% (*n* = 531) originated from France and 11.1% (*n* = 106) from the United States. Of note, the proportion of reports classified as IMEs was high with concomitant 5-HT3 RA and NK-1 RA therapy: 64% (*n* = 554) of ADE cases were classified as IMEs.

**Figure 3:**
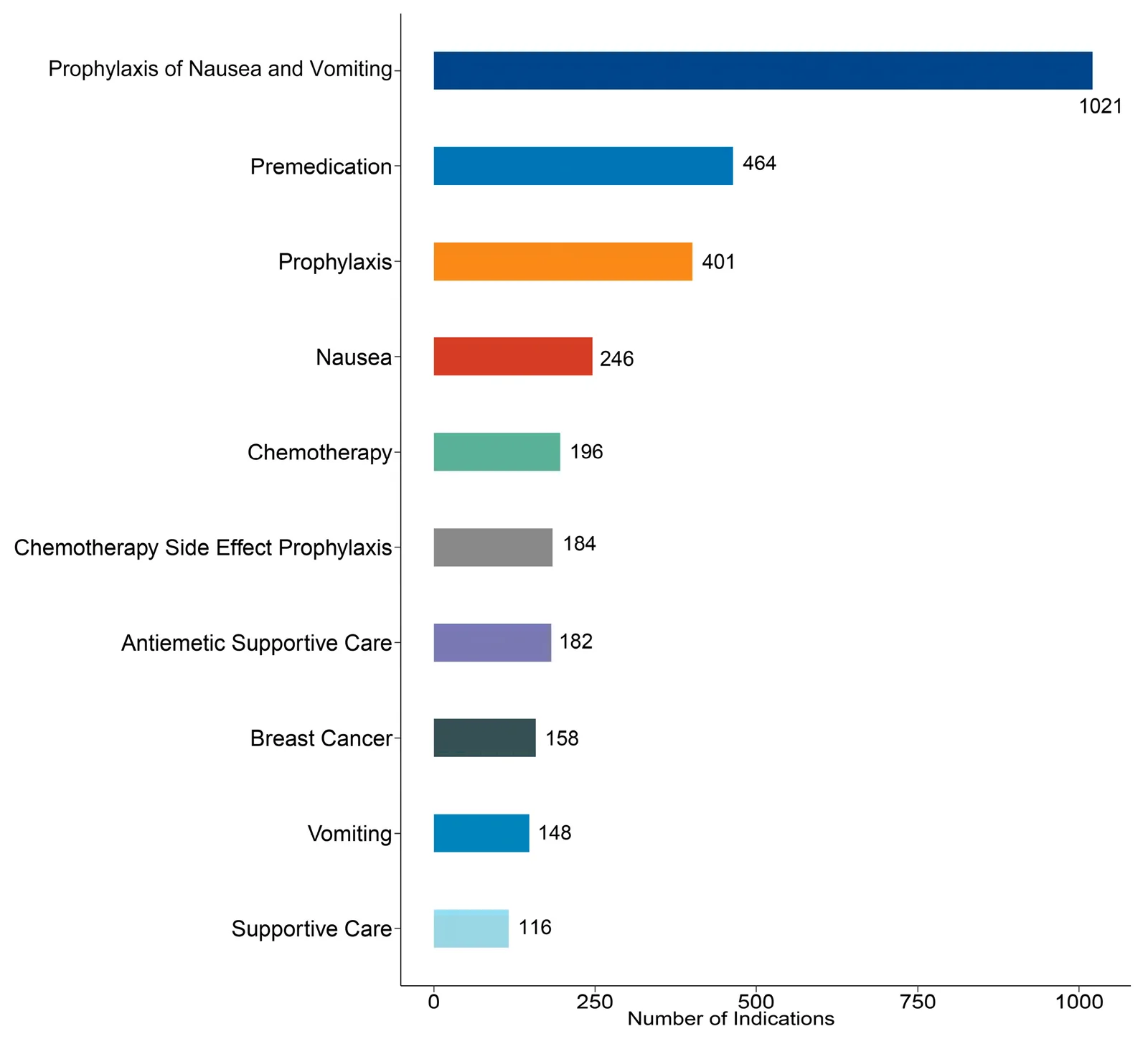
Statistical bar chart of indications for Combined Use of 5-HT3 RA and NK-1 RA. Indication categories are not mutually exclusive; a single report may contribute to more than one indication term. 5-HT3 RA, 5-Hydroxytryptamine Type 3 Receptor Antagonist; NK-1 RA, neurokinin-1 Receptor Antagonist.

We then summarized all indications for concomitant 5-HT3 RA and NK-1 RA use; the Indication PTs and corresponding report counts are provided in Table S1. After excluding “Product Used for Unknown Indication,” the top ten indications were: Prophylaxis of Nausea and Vomiting; Premedication; Prophylaxis; Nausea; Chemotherapy; Chemotherapy Side Effect Prophylaxis; Antiemetic Supportive Care; Breast Cancer; Vomiting; and Supportive Care. Visualization (Figure 3) indicates that this combination is most frequently employed for the prevention of CINV. Furthermore, combination therapy statistics show that 5-HT3 RAs and NK-1 RAs are generally used in combination with chemotherapy drugs and other drugs for the prevention of CINV (Table S2).

Collectively, these findings indicate that ADEs reported with concomitant 5-HT3 RA and NK-1 RA use warrant heightened attention. Vigilant evaluation of combination regimens in clinical practice is essential and underpins comprehensive, longitudinal management of affected patients.

### 2.2. Signal Detection Analysis

In the signal-detection analysis, we applied five algorithms to 366 PTs; full results are provided in Table S3. For clarity, 965 refers to unique concomitant-use reports, 3374 (*n*_11+_) refers to the total number of adverse-event records across all preferred terms, and 366 refers to the number of distinct preferred terms evaluated. To ensure robustness, we retained PTs significant across all five algorithms and, after excluding terms unrelated to drug-induced events (e.g., disease entities), identified 49 PTs (Table 2). The most frequently reported were Erythema (*n* = 87), Hypersensitivity (*n* = 78), Hypotension (*n* = 48), Hyponatraemia (*n* = 37), Hypertension (*n* = 37), Acute Kidney Injury (*n* = 33), Encephalopathy (*n* = 29), Seizure (*n* = 24), Hepatocellular Injury (*n* = 22), and White Blood Cell Count Decreased (*n* = 20). The data indicate that ADE signals observed in reports involving concomitant 5 HT3 RAs and NK 1 RAs primarily involved disorders of the skin and subcutaneous tissue, hypersensitivity reactions, blood-pressure abnormalities, hepatic and renal injury, and neurological disturbances. Notably, we also identified significant PTs not highlighted in the product labeling for 5-HT3 RAs and NK-1 RAs—such as abnormal antidiuretic hormone secretion, respiratory disorders, and oropharyngeal disorders—that were disproportionately reported and warrant consideration in clinical practice.

### 2.3. Sensitivity Analysis and Subgroup Analysis

Sensitivity analysis yielded similar results; we still retained only 85 PTs that were significant across all five algorithms (Table S4). The PTs with the most reported cases were Dyspnoea (*n* = 210), Pyrexia (*n* = 202), Erythema (*n* = 177), Cough (*n* = 113), Hypotension (*n* = 106), Acute Kidney Injury (*n* = 103), Hyponatraemia (*n* = 102), Atelectasis (*n* = 102), Hypoxia (*n* = 88), and Hepatocellular Injury (*n* = 87). In the subgroup analysis, we stratified participants by age, body weight, and sex to investigate ADEs associated with concomitant 5-HT3 RAs and NK-1 RAs.

**Figure 4:**
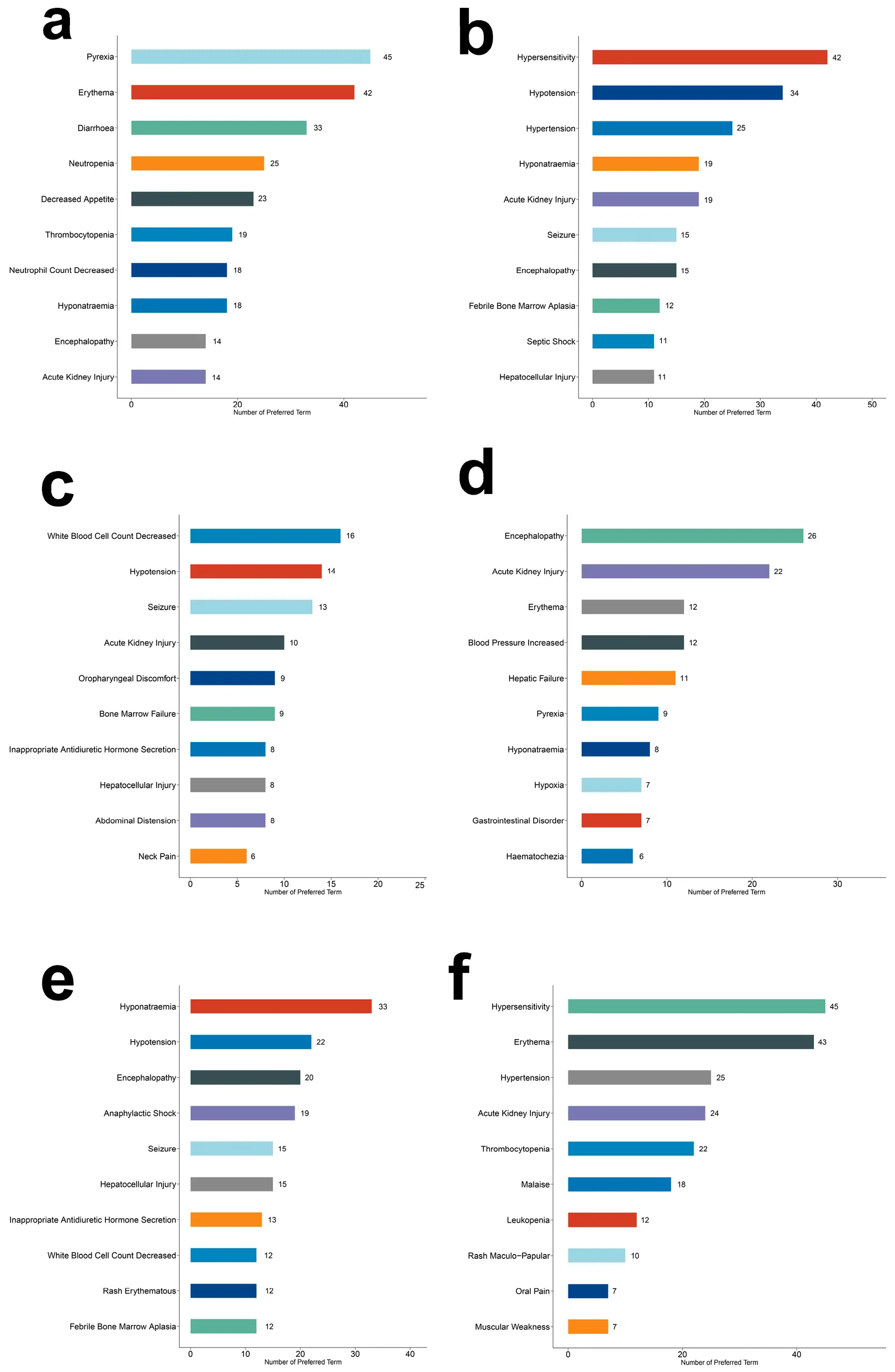
(**a**) In the 18–59 age subgroup; (**b**) in the ≥60 age subgroup; (**c**) in the weight subgroup less than 67 kg; (**d**) in the weight subgroup greater than or equal to 67 kg; (**e**) in the female subgroup; (**f**) in the male subgroup, all five algorithms were significant and the number of PT reports ranked in the top 10.

We again retained PTs that exceeded the threshold across all five algorithms and visualized the ten PTs with the highest report counts. Among those aged 18–59 years (Figure 4a), Pyrexia (*n* = 45) was most frequently reported; among those aged ≥60 years (Figure 4b), Hypersensitivity (*n* = 42) predominated, with blood-pressure abnormalities also common. In the lower-weight cohort (Figure 4c), White Blood Cell Count Decreased (*n* = 16) was most frequent; in the higher-weight cohort (Figure 4d), Encephalopathy (*n* = 26) ranked highest. Among women (Figure 4e), Hyponatraemia (*n* = 33) and Hypotension (*n* = 22) were more frequently reported; among men (Figure 4f), Hypersensitivity (*n* = 45) and Erythema (*n* = 43) were more commonly reported. Notably, Hypertension (*n* = 25) was also more frequently reported in men than in women; because no formal interaction test or direct statistical comparison was performed, these subgroup findings represent descriptive reporting frequencies rather than evidence of sex-specific differences. Importantly, with the exception of women, Acute Kidney Injury ranked among the top ten significant PTs across the other five subgroups, highlighting the need to monitor renal function during concomitant 5-HT3 RAs and NK-1 RAs therapy.

### 2.4. Probability Density Function of Time-to-Onset

**Figure 5:**
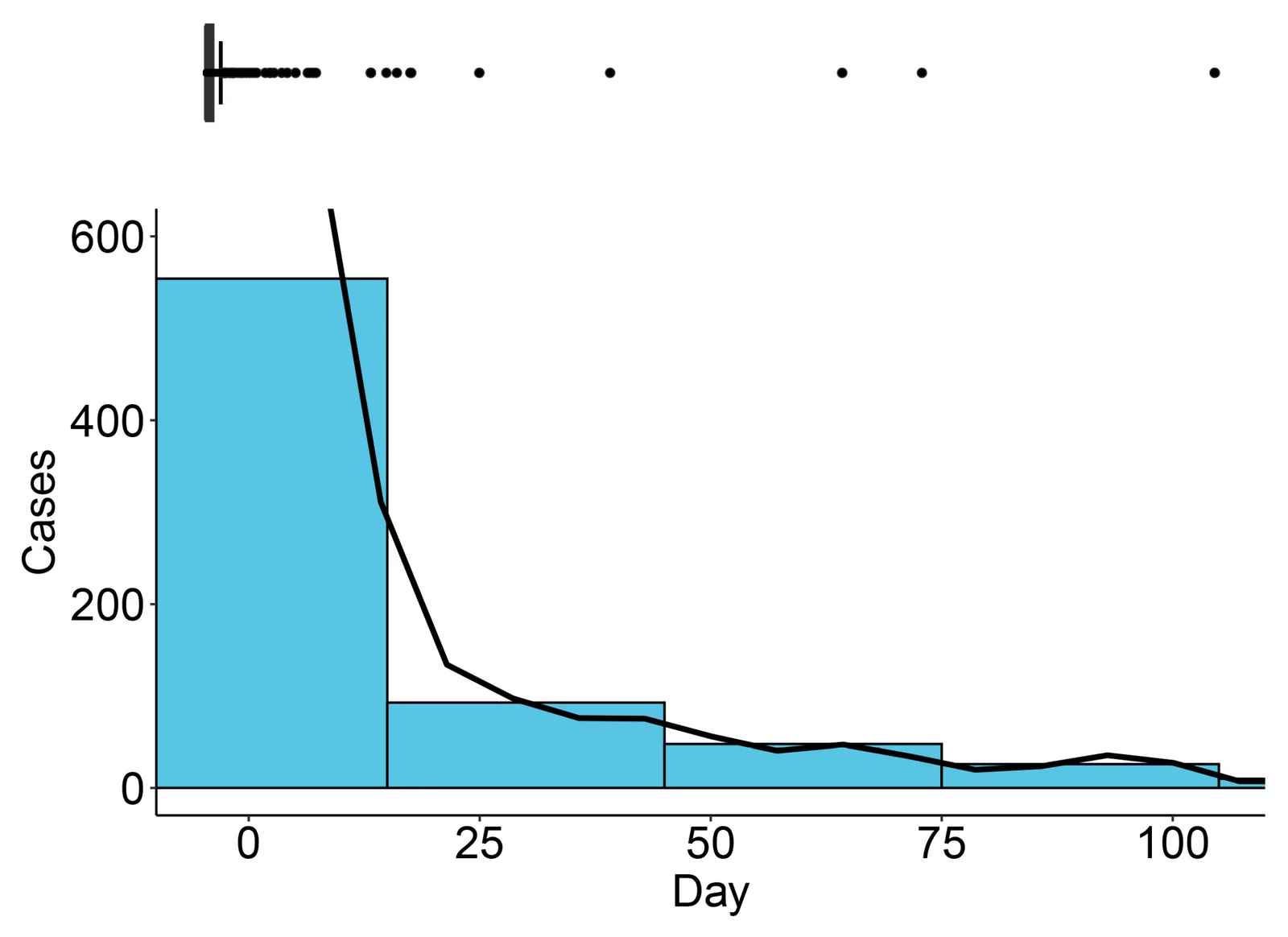
The frequency distribution of ADE occurrence based on time-to-onset (combined histogram, estimated density, and boxplot; *n* = 778). ADE, adverse drug event.

To characterize the timing of ADEs associated with concomitant 5-HT3 RAs and NK-1 RAs, we visualized TTO using histogram–density–boxplots (Figure 5). The distribution shows a steep decline in event counts over time, with the vast majority occurring within 20 days of treatment initiation. Accordingly, we observed that reported onset times were concentrated in the early period after starting concomitant therapy.

A Weibull analysis (Table 3) was consistent with this pattern: the median TTO was 4 days, and the shape parameter α was <1 with the upper bound of its 95% CI below 1, indicating a concentration of reported onset times in the early treatment period.

### 2.5. Logistic Regression for the Occurrence of IMEs

In prior descriptive analyses, 64% of ADE cases with known outcome information were classified as IMEs. Accordingly, we fitted univariable and multivariable logistic regression models to examine associations between age, body weight, sex, and IME occurrence; detailed parameters are reported in Table 4. In the univariable model, age (continuous) was significantly associated with IMEs (OR = 1.01, 95% CI: 1.00–1.02, *P* = 0.013); participants aged ≥60 years had 1.34-fold higher odds than those aged 18–59 years (OR = 1.34, 95% CI: 1.04–1.73, *P* = 0.026). In the multivariable model adjusting for body weight and sex, age remained significant: each 1-year increase was associated with 4% higher odds of IME (OR = 1.04, 95% CI: 1.02–1.06, *P* < 0.001), and adults ≥60 years had 2.45-fold higher odds than those aged 18–59 (OR = 2.45, 95% CI: 1.72–3.49, *P* < 0.001). Body weight and sex were not significantly associated with IMEs in either model. These findings suggest that older age was associated with higher odds of IME classification among the included reports, warranting heightened vigilance in older patients receiving concomitant 5-HT3 RAs and NK-1 RAs.

## 3. Discussion

CINV arises through two principal mechanisms. Approximately 95% of 5-HT is synthesized, stored, and released by enterochromaffin cells of the intestinal mucosa and by myenteric neurons [6,7]. Peripheral 5-HT3-mediated pathways originating in the gastrointestinal tract are activated within the first 24 h after chemotherapy and primarily drive acute emesis, whereas central NK-1-mediated pathways in the brain are believed to underlie delayed CINV [8]. Accordingly, 5-HT3 RAs and NK-1 RAs are the mainstays of CINV prophylaxis. The FDA approved the 5-HT3 RA ondansetron in 1991 for the prevention of chemotherapy-induced emesis, followed in 1997 by approvals of granisetron and dolasetron for the same indication. 5-HT3 RAs share structural similarity with 5-HT and bind the same site on the 5-HT3 receptor, thereby preventing its activation. Substance P (SP), a neurotransmitter present in both the peripheral and central nervous systems, is also released during chemotherapy and binds NK-1 receptors, contributing to CINV; in 2003, the NK-1 RA aprepitant was approved [9]. Since then, combination prophylaxis with 5-HT3 RAs and NK-1 RAs has marked a milestone in preventing CINV. However, some adverse reactions have been recorded in the long term, and the potential pharmacokinetic interaction mechanisms between NK-1 RAs and 5-HT3 RAs remain unclear. Given their widespread concomitant use and these safety considerations, systematically characterizing potential safety signals associated with their concomitant use in a large spontaneous reporting system is clinically pertinent.

To investigate adverse event reporting patterns associated with the concomitant use of 5-HT3 RAs and NK-1 RAs, we extracted FAERS data from Q1 2004 through Q1 2025 and identified 965 reports involving concomitant use. Summary statistics indicate that reports of ADEs with the combination are increasing and that the proportion of reports classified as IMEs is high, underscoring the clinical importance of delineating these reporting patterns to support safe use. With respect to indications and combination therapy statistics, concomitant 5-HT3 RAs and NK-1 RAs were used predominantly for CINV, consistent with clinical practice.

Next, we assessed reporting patterns associated with concomitant 5-HT3 RA and NK-1 RA use using five methods: the Ω shrinkage measure, additive model, multiplicative model, combination risk ratio, and Chi-square model. The results indicate that several adverse events—including neurological disorders, skin and subcutaneous tissue reactions, hypersensitivity reactions, blood-pressure abnormalities, and hepatic and renal injury—were disproportionately reported with concomitant use. Meanwhile, the results of the sensitivity analysis enhanced the robustness of the above findings.

The sensitivity analysis that additionally included reports in which the target drugs were coded as secondary suspects was performed to test whether the primary signal set was robust to a broader exposure definition. The appearance of additional, often more systemic events such as Dyspnoea and Pyrexia in this analysis reflects the inclusion of a larger and more heterogeneous report population. This indicates that the primary analysis, restricted to primary-suspect drugs, is more specific, whereas the sensitivity analysis is more sensitive but less specific. The concordance of the core signal set across both approaches supports the robustness of the main findings.

As is well recognized, 5-HT is a key neurotransmitter whose signaling perturbations are closely linked to neuropsychiatric disease; it modulates multiple targets, most prominently hypothalamic, limbic, and cortical circuits governing mood and affect control [10]. Dysregulated serotonergic signaling has been implicated in anxiety, depression, and related psychiatric conditions [11,12]. Most studies suggest that enhancing 5-HT function can attenuate seizures and reduce the risk of sudden unexpected death in epilepsy [13]; standard antiepileptic drugs (e.g., valproate, carbamazepine, phenytoin, lamotrigine, and zonisamide) may enhance 5-HT release, contributing to anticonvulsant effects [14,15,16], whereas depletion of brain 5-HT facilitates seizure induction [17,18]. Notably, generalized tonic–clonic seizures have been reported after intravenous palonosetron [19]. On the other hand, SP is widely distributed in the central and peripheral nervous systems; upon binding NK-1 receptors, SP modulates many central nervous system pathophysiological processes—affective behavior, stress, depression, anxiety, emesis, migraine, alcohol addiction, seizures, and neurodegeneration [20]. The SP/NK-1 receptor system may precipitate seizures and plays a key role in status epilepticus and experimental epilepsy models [21,22,23]. Thus, concomitant use may be linked to an increased reporting of seizures and other neurological events; this potential association should be further explored in mechanistic or prospective studies.

5-HT is widely recognized not only as a neurotransmitter but also as an immunomodulator in the periphery, including the skin [24]. It plays a pivotal role in immune regulation, particularly in recruiting immune cells to inflammatory sites [25]; dysregulated 5-HT signaling has been linked to multiple inflammatory dermatoses, with significantly more 5-HT-positive cells in inflamed than in normal skin [26]. Meanwhile, SP and NK receptors are key mediators of visceral hypersensitivity [27,28]; in mice, NK-1 receptor signaling has been implicated in pruritus [29]. Although NK-1 RAs are being explored as potential therapies for chronic dermatoses [30,31], injection-site reactions are common ADEs with NK-1 RAs [32], and IV rolapitant was withdrawn by the FDA because of hypersensitivity and allergic reactions [33]. In our study, significant drug-drug interactions (DDIs) signals for the combination included not only Erythema (*n* = 87) and Hypersensitivity (*n* = 78), but also Rash Maculo-Papular (*n* = 15), Rash Erythematous (*n* = 15), Toxic Epidermal Necrolysis (*n* = 12), Mucosal Inflammation (*n* = 11), Rash Pruritic (*n* = 10), and Palmar–Plantar Erythrodysaesthesia Syndrome (*n* = 7). Collectively, cutaneous abnormalities and hypersensitivity were among the most frequently reported adverse events with concomitant 5-HT3 RAs and NK-1 RAs, a pattern that may reflect peripheral immune dysregulation secondary to disturbances in 5-HT and SP/NK-1 signaling.

Regarding blood pressure, activation of 5-HT3 receptors can stimulate the vagus nerve [34], potentially eliciting reflex bradycardia and hypotension. Ondansetron has been reported to raise blood pressure in rats [35] and to ameliorate spinal-anesthesia-induced hypotension when given intravenously [36,37]; nonetheless, hypotension is listed as an uncommon reaction in the ondansetron label. Although 5-HT broadly participates in cardiovascular regulation, its physiological role in vascular tone remains incompletely defined [38]. NK-1 receptors contribute to blood-pressure control through vasodilation and modulation of central sympathetic outflow, with activation generally lowering blood pressure; prior work indicates that SP and NK receptors influence blood-pressure regulation [39,40]. Neurokinin A-induced hypotension can occur independently of the vagus nerve, likely via direct action on vascular smooth muscle expressing NK-1 receptors [41], whereas central NK-1 receptors may help maintain mean arterial pressure after high-level spinal cord injury [42]. In sum, our findings suggest that blood-pressure abnormalities were disproportionately reported with concomitant use of the two agents, warranting further investigation and underscoring the need for blood-pressure monitoring during concomitant use.

Beyond labeled events, we detected additional ADEs, notably inappropriate antidiuretic hormone secretion, likely reflecting neuroendocrine involvement. 5-HT is an important regulator of pituitary hormone production and hypothalamic–pituitary–adrenal axis activity [11]; NK-1 receptors are present in the nervous system and peripheral tissues, participating in endocrine and paracrine processes [43], and the SP/NK-1 system can directly stimulate mineralocorticoid synthesis and influence salt–water balance [44]. Accordingly, Inappropriate Antidiuretic Hormone Secretion and hyponatraemia may be mechanistically linked. Aprepitant can attenuate SP-mediated vasorelaxation, reducing aldosterone production by approximately 30% in healthy men and decreasing adrenal blood flow [44], which may also contribute to acute kidney injury. In respiratory control, both 5-HT and SP participate in the neural regulation of breathing [45]; serotonergic effects may be mediated at the medullary level [46], and 5-HT has been proposed as a biomarker for obstructive sleep apnea [47]. SP and NK receptors in airway sensory nerves modulate tracheal smooth-muscle tone; in intact rats, intravenous NKA transiently increases respiratory rate while decreasing tidal volume, followed by prolonged respiratory depression with sustained increases in tidal volume [41]. Notably, ondansetron has been associated with dysphagia [48]; in our dataset, we also observed 11 ADE reports of Hypophagia, with significance across all five algorithms. Subgroup analyses yielded concordant results.

To make these findings actionable for clinicians, we prioritized the signals that were both disproportionately reported and least well recognized from the individual product labels or from the expected toxicity of chemotherapy. The most clinically relevant novel or concerning signals were (i) inappropriate antidiuretic hormone secretion and hyponatraemia, which support electrolyte monitoring [49]; (ii) oropharyngeal disorders and related mucosal events, which should be distinguished from chemotherapy- and radiotherapy-induced mucositis [50]; (iii) respiratory events, which warrant vigilance in patients with pre-existing pulmonary disease; (iv) blood-pressure abnormalities, supporting routine blood-pressure assessment; and (v) hypersensitivity and severe cutaneous reactions, for which patients should be counselled to report early skin changes. By contrast, haematological events (for example, decreased white blood cell count and febrile bone marrow aplasia), acute kidney injury, and septic shock should be interpreted primarily as manifestations of the underlying malignancy and its treatment rather than as effects of the antiemetics [51]. A tiered monitoring approach is therefore suggested: first-tier monitoring of electrolytes, blood pressure, and early signs of hypersensitivity; second-tier surveillance for respiratory and oropharyngeal symptoms; and continued routine monitoring of haematological and renal parameters according to the chemotherapy protocol.

Our TTO analysis indicated a median onset of 4 days, with the majority of events occurring within 20 days of initiating concomitant antiemetic therapy. And the Weibull analysis showed that reported onset times were concentrated in the early period, emphasizing the need for early clinical surveillance. Moreover, whether modeled continuously or categorically, age consistently emerged as a factor associated with IME classification in both univariable and multivariable logistic regression, indicating that greater caution is warranted in older adults. Clinically, this suggests that the highest vigilance window is the first week after starting 5-HT3 RAs plus NK-1 RAs, extending through the initial 2–3 weeks. Focused monitoring during this period—particularly in older adults, in whom higher odds of IME classification were observed—may enable earlier detection and mitigation of clinically meaningful adverse events.

The concentration of reported onset times within approximately 20 days of starting the antiemetic combination is consistent with the time course of chemotherapy. Acute CINV typically develops within the first 24 h and delayed CINV within the following days, and the period of greatest myelosuppression and organ toxicity also occurs in the first few weeks [52]. Consequently, some of the early events reported with concomitant use may represent a bystander effect in which patients are at highest risk during the period of maximal chemotherapy toxicity and supportive-care exposure, rather than a direct pharmacological effect of the antiemetics. This possibility, together with protopathic bias and reporting practices, further cautions against interpreting the temporal pattern as evidence of a true decline in risk.

This real-world pharmacovigilance study explored reported adverse event patterns associated with concomitant 5 HT3 RAs and NK 1 RAs, identifying potential safety signals that merit further clinical investigation. Nonetheless, given data limitations, we included only two classes of antiemetics; other agents commonly used in combination regimens, such as olanzapine and dexamethasone, were not incorporated, and residual confounding cannot be excluded [53]. In particular, most patients in this dataset were receiving cytotoxic chemotherapy for an underlying malignancy, and events such as decreased white blood cell count, febrile bone marrow aplasia, septic shock, and acute kidney injury are well-recognized consequences of cancer and anticancer treatment rather than of the antiemetics themselves. Because FAERS does not reliably capture chemotherapy regimens or their intensity, this confounding is a central limitation that cannot be resolved in the present analysis. Inherent constraints of spontaneous-reporting systems—unknown biases not fully mitigable by statistical methods—introduce irreducible error. The database’s incompleteness also precludes definitive causal inferences between the DDI and related ADEs. Our study detected reporting signals rather than conducted a risk assessment; for example, reports of White Blood Cell Count Decreased likely reflect concomitant chemotherapy. Despite these limitations, comprehensive analyses leveraging the world’s largest pharmacovigilance resource remain highly informative. In clinical practice, individual context should guide decisions—for example, vigilance for seizures and other encephalopathies in patients with neurologic disease—and vital signs should be closely monitored, with periodic reassessment of hepatic and renal function and electrolytes before, during, and after therapy.

Several additional limitations should be considered. First, palonosetron and netupitant were not included because, in contemporary practice, they are predominantly marketed as a fixed-dose oral combination, which is recorded as a single product and therefore cannot be decomposed into its individual components for the drug–drug interaction algorithms used here [54]. Second, 55.5% of reports originated from France, which may reflect country-specific reporting practices or product utilization and could influence the observed signal distribution. A sensitivity analysis excluding French reports was not performed in this revision, so it remains unknown whether the principal signals are robust to this substantial country-specific contribution; this should be examined in future work. Third, we evaluated 366 preferred terms without a formal false-discovery-rate adjustment. We retained the prespecified requirement for concordance across all five algorithms because it is a conservative selection rule that substantially reduces the candidate-signal set (49 of 366 terms) and prioritizes specificity; however, concordance across the five statistically related algorithms should not be interpreted as equivalent to multiplicity correction, and signals based on very small numbers of reports (three to five cases) should be interpreted with particular caution. Finally, as with all spontaneous-reporting analyses, the findings represent disproportionate reporting patterns rather than incidence estimates or confirmed interactions [53].

## 4. Materials and Methods

### 4.1. FAERS Data Curation and Refinement

We extracted publicly accessible individual ADE reports from FAERS, submitted by healthcare professionals, consumers, and manufacturers. The FAERS dataset comprises seven tables: patient demographic and administrative information (DEMO), drug/biologic information (DRUG), adverse events (REAC), patient outcomes (OUTC), report sources (RPSR), drug therapy start and end dates (THER), and indications for use/diagnosis (INDI). We included 5-HT3 RAs and NK-1 RAs, encompassing six 5-HT3 RAs (Ondansetron, Granisetron, Tropisetron, Azasetron, Ramosetron, Dolasetron) and three NK-1 RAs (Aprepitant, Fosaprepitant, Rolapitant); all search terms are listed in Table S5. We retrieved all reports dated 1 January 2004–31 March 2025 and deduplicated them per FDA guidance [55,56,57]. The analysis was restricted to reports involving adult patients (aged ≥18 years); reports involving patients younger than 18 years were excluded. In FAERS, drug roles are coded as primary suspected (PS), second suspected (SS), concomitant (C), and interacting (I); to ensure reliability, we analyzed only reports in which the target drugs were coded as PS, excluding SS, C, and I. Reports containing more than one drug within the same class were counted once per drug class at the report level. ADEs were coded using MedDRA v28.0 (https://www.meddra.org/) [58,59], and analyses were conducted at the preferred term (PT) level. All statistical analyses and visualizations were performed in R v4.4.3.

### 4.2. Descriptive Analysis

After screening ADEs reports involving concomitant therapy, we first conducted a descriptive analysis of baseline report characteristics, including demographics (sex, age, weight), reporter occupation (OCCP_COD), reporting country (REPORTER_COUNTRY), clinical outcomes (OUTC_COD), and reporting year. We also paid particular attention to the indications for concomitant 5-HT3 RAs and NK-1 RAs use in FAERS, as well as the combination therapy.

### 4.3. Drug–Drug Interaction Signal Analysis

To identify ADEs associated with concomitant 5-HT3 RA–NK-1 RA use, we applied five algorithms: the Ω shrinkage measure, additive model, multiplicative model, combination risk ratio, and chi-square model [60]. Signals exceeding the algorithm-specific threshold were interpreted as indicating a potential association between drug exposure and the ADEs. All five algorithms are based on a 4 × 2 contingency table; detailed formulas and thresholds are provided in Table 5. As a conservative selection rule, we deemed an ADE signal significant only if it surpassed the threshold across all five algorithms; concordance across the five measures does not constitute independent validation, because all are derived from the same 4 × 2 contingency table.

(1) Ω shrinkage measure model:


(1) $$\Omega_{025}=\Omega -\frac{\phi (0.975)}{\log(2)\sqrt{n_{111}}}>0$$

(2) Additive model:


(2) $$\frac{n_{111}}{n_{11+}}-\frac{n_{101}}{n_{10+}}-\frac{n_{011}}{n_{01+}}-\frac{n_{001}}{n_{00+}}>0$$

(3) Multiplicative model:


(3) $$\frac{n_{111}\times n_{001}\times n_{10+}\times n_{01+}}{n_{11+}\times n_{00+}\times n_{101}\times n_{011}}>1$$

(4) Combination risk ratio model:


(4) $$CRR=\frac{PRR_{DrugA\cap DrugB}}{\max\left(PRR_{DrugA},PRR_{DrugB}\right)}>2\;n_{111}\geq 3,\;PRR_{DrugA\cap DrugB}>2,\;\chi_{DrugA\cap DrugB}^{2}>4$$

(5) Chi-Square statistics model:


(5) $$\chi =\frac{n_{111}-E_{111}-0.5}{\sqrt{E_{111}}}>2$$

### 4.4. Sensitivity Analysis and Subgroup Analysis

To improve the robustness of the results and reduce background noise, we included ADE reports collected from drugs reported as PS and SS for sensitivity analysis. On the other hand, FAERS provides three demographic variables—sex, age, and weight. To assess differences in ADEs associated with concomitant use of the two drugs across populations, we conducted subgroup disproportionality analyses based on these demographics. Using the same five algorithms, we stratified by age (18–59 years, ≥60 years), weight (<67 kg, ≥67 kg), and sex (female, male); the 67 kg cutoff approximated the median weight among reports with available data.

### 4.5. Probability Density Function of Time-to-Onset

Time to onset (TTO) was defined as the interval between treatment initiation and ADE onset. We included cases with complete, valid dates for TTO analysis and summarized TTO using the median, interquartile range, and range. Of the 965 reports, 778 had complete and valid dates and were included in the TTO analysis; the remaining reports were excluded because of missing, incomplete, inconsistent, or implausible dates. TTO was computed from the earliest recorded treatment-start date available in each record, and repeated administration across chemotherapy cycles could not be reliably distinguished; consequently, the maximum reported TTO of 3653 days may reflect data-entry errors, repeated cycles, or use of the first recorded treatment date. TTO values of zero (events reported on the same day as treatment initiation) were treated as an extremely short positive time interval to permit Weibull fitting, because the standard Weibull distribution is defined only for positive event times. We modeled the TTO probability density with a Weibull distribution, which can capture increasing or decreasing risk over time. The Weibull distribution is parameterized by a shape parameter α (>0) and a scale parameter β (>0). The probability density function formula is provided in Table S6. In this study, we focused on α; when α < 1 and the upper bound of its 95% CI is below 1, reported onset times were concentrated in the early treatment period (early-failure-type pattern); when α = 1, reported onset times were approximately constant over time (random-failure); when α > 1 and the upper 95% CI bound exceeds 1, reported onset times increased over time (wear-out failure). These patterns describe the distribution of reported onset times rather than a demonstrated change in the underlying hazard, because FAERS does not contain event-free follow-up or exposure denominators.

### 4.6. Logistic Regression

FAERS categorizes ADE outcomes into seven groups—congenital anomaly (CA), death (DE), disability (DS), hospitalization—initial or prolonged (HO), life-threatening (LT), required intervention to prevent permanent impairment/damage (RI) and other (OT)—of which CA, DE, DS, HO, RI, and LT are classified as important medical events (IME); reports with an “Other” (OT) outcome were treated as the non-IME reference group, although an “Other” outcome does not necessarily indicate a clinically non-serious event. We evaluated associations between age, weight, and sex and the occurrence of IME using univariate and multivariable logistic regression. The adjustment set was limited to age, weight, and sex because information on chemotherapy regimen and intensity, cancer type, and other concomitant medications was not available in the extracted records; the model therefore cannot account for these important sources of confounding. Reports with missing outcome information were excluded from the logistic regression models, and the IME proportion of 64% reflects the 865 reports with known outcome information. Because the univariable and multivariable models were fitted on different complete-case samples, the AIC values reported in Table 4 are not directly comparable across models. In the multivariable models, each variable was entered as the primary predictor with the other two as covariates; age and weight were modeled as continuous variables. Statistical significance was defined as a two-sided *P* < 0.05.

## 5. Conclusions

Overall, we analyzed FAERS reports spanning more than two decades to characterize adverse event reporting patterns associated with the concomitant use of 5-HT3 RAs and NK-1 RAs. Methodologically, we applied five disproportionality-based algorithms, a more comprehensive approach than conventional single-method analyses. To our knowledge, this is the first FAERS-based evaluation of adverse event reporting patterns associated with the concomitant use of these two antiemetic classes. Our findings suggest that the number of reports of ADEs with this combination is increasing, highlighting a need for ongoing surveillance. In clinical practice, concomitant use warrants heightened vigilance for skin and subcutaneous tissue disorders, hypersensitivity reactions, blood-pressure abnormalities, and hepatic and renal injury, while recognizing that many of these events may reflect the underlying cancer and its chemotherapy. Moreover, abnormal antidiuretic hormone secretion, respiratory disorders, and oropharyngeal disorders—signals not described in product labeling—deserve attention. Reported onset times were concentrated in the early period after therapy initiation, and older age was associated with higher odds of IME classification among the included reports. These findings are hypothesis-generating and require confirmation in prospective studies.

## Figures and Tables

**Table 1:** Demographic characteristics of 965 ADE cases.

Characteristics	*N* = 965 ^1^
Age	58 (13)
18–59 years	467 (48.4%)
>=60 years	498 (51.6%)
Weight	70 (18)
<67 kg	272 (49.6%)
>=67 kg	276 (50.4%)
Unknown	417
Sex	
Female	575 (59.7%)
Male	388 (40.3%)
Unknown	2
Reported person	
Health professional—Physician (MD)	344 (36.2%)
Non-healthcare professional—Consumer (CN)	73 (7.7%)
Pharmacist (PH)	189 (20.0%)
Health professional (HP)	101 (10.6%)
Other health professional (OT)	242 (25.5%)
Unknown	16
Reported country	
France	531 (55.5%)
America	106 (11.1%)
United Kingdom	83 (8.7%)
China	47 (4.9%)
Korea	38 (4.0%)
Canada	35 (3.7%)
Japan	24 (2.5%)
Netherlands	22 (2.3%)
Germany	14 (1.5%)
Lithuania	8 (0.8%)
Spain	7 (0.7%)
Switzerland	5 (0.5%)
Norway	5 (0.5%)
Czech Republic	4 (0.4%)
Greece	4 (0.4%)
Luxembourg	4 (0.4%)
Australia	3 (0.3%)
Brazil	3 (0.3%)
Denmark	3 (0.3%)
Portugal	3 (0.3%)
Finland	2 (0.2%)
Russia	2 (0.2%)
India	1 (0.1%)
Italy	1 (0.1%)
Sweden	1 (0.1%)
Unknown	9
Outcome	
Hospitalization—Initial or prolonged (HO)	388 (44.9%)
Other (OT)	311 (36.0%)
Death (DE)	88 (10.2%)
Life-threatening (LT)	77 (8.9%)
Required intervention to prevent permanent impairment/damage (RI)	1 (0.1%)
Unknown	100

^1^ Mean (SD); *N* (%).

**Table 2:** Results of signal detection analysis of significant ADE signals.

PT	*n*_111_	*n*_11+_	*E*_111_	Ω (Ω_025_)	AM	MM	CRR	χ
Erythema	87	3374	50.09	0.79 (0.49)	0.0109	4.24	2.20	5.15
Hypersensitivity	78	3374	43.53	0.83 (0.51)	0.0102	4.48	2.24	5.15
Hypotension	48	3374	22.64	1.07 (0.66)	0.0077	7.18	2.12	5.23
Hyponatraemia	37	3374	8.87	2.00 (1.54)	0.0083	4.60	4.31	9.28
Hypertension	37	3374	21.49	0.77 (0.30)	0.0046	2.75	2.09	3.24
Acute Kidney Injury	33	3374	13.26	1.28 (0.79)	0.0092	3.57	3.70	5.28
Encephalopathy	29	3374	12.87	1.14 (0.62)	0.0048	3.92	2.43	4.36
Seizure	24	3374	13.55	0.80 (0.22)	0.0031	2.88	2.19	2.70
Hepatocellular Injury	22	3374	6.01	1.79 (1.19)	0.0047	37.32	4.57	6.32
White Blood Cell Count Decreased	20	3374	9.07	1.10 (0.47)	0.0039	1.51	2.20	3.46
Bone Marrow Failure	17	3374	3.82	2.02 (1.33)	0.0041	2.06	4.45	6.49
Oral Pain	17	3374	3.82	2.02 (1.33)	0.0041	2.67	4.45	6.49
Rash Maculo-Papular	15	3374	5.28	1.42 (0.69)	0.0032	109.69	2.84	4.01
Rash Erythematous	15	3374	6.92	1.06 (0.33)	0.0027	27.75	2.17	2.88
Inappropriate Antidiuretic Hormone Secretion	13	3374	3.59	1.72 (0.94)	0.0028	5.27	3.89	4.70
Leukopenia	12	3374	5.28	1.11 (0.30)	0.0022	8.55	2.27	2.71
Febrile Bone Marrow Aplasia	12	3374	1.30	2.79 (1.98)	0.0032	115.84	11.39	8.93
Toxic Epidermal Necrolysis	12	3374	4.46	1.33 (0.52)	0.0024	116.50	2.69	3.34
Dysarthria	12	3374	3.99	1.48 (0.66)	0.0028	20.33	3.01	3.76
Hot Flush	11	3374	5.52	0.93 (0.08)	0.0016	2.24	2.09	2.12
Mucosal Inflammation	11	3374	3.79	1.42 (0.57)	0.0021	4.33	3.29	3.44
Hypophagia	11	3374	1.91	2.25 (1.40)	0.0028	4.40	5.76	6.22
Septic Shock	11	3374	3.34	1.58 (0.73)	0.0024	2.75	3.29	3.91
Cholestasis	10	3374	2.93	1.61 (0.72)	0.0024	44.44	3.41	3.84
Rash Pruritic	10	3374	3.87	1.26 (0.37)	0.0023	8.40	2.58	2.86
Cyanosis	9	3374	4.08	1.05 (0.11)	0.0015	7.45	3.14	2.18
Interstitial Lung Disease	9	3374	3.34	1.31 (0.36)	0.0021	1.51	2.69	2.82
Mouth Ulceration	9	3374	1.43	2.30 (1.35)	0.0024	2.95	6.29	5.90
Oropharyngeal Discomfort	9	3374	2.60	1.61 (0.67)	0.0019	21.95	5.48	3.65
Respiratory Distress	8	3374	3.40	1.12 (0.12)	0.0014	12.26	2.35	2.22
Peritonitis	8	3374	1.24	2.29 (1.29)	0.0024	9.53	11.38	5.63
Jaundice	7	3374	2.35	1.40 (0.33)	0.0016	9.53	2.98	2.71
Blood Alkaline Phosphatase Increased	7	3374	1.76	1.73 (0.66)	0.0019	15.06	3.98	3.57
Palmar–Plantar Erythrodysaesthesia Syndrome	7	3374	2.39	1.38 (0.31)	0.0016	1.20	2.93	2.66
Acute Respiratory Failure	7	3374	1.31	2.05 (0.99)	0.0021	1.98	7.33	4.55
Large Intestine Perforation	7	3374	1.44	1.95 (0.88)	0.0016	46.25	4.97	4.22
Gamma-Glutamyltransferase Increased	6	3374	1.91	1.43 (0.28)	0.0012	3.06	3.14	2.60
Sense Of Oppression	6	3374	1.22	1.92 (0.76)	0.0014	25.83	6.31	3.87
Ear Pain	6	3374	2.39	1.17 (0.02)	0.0012	1.49	2.51	2.01
Sputum Discoloured	6	3374	0.62	2.53 (1.38)	0.0018	4.72	12.58	6.17
Epilepsy	6	3374	2.07	1.34 (0.18)	0.0012	3.46	3.14	2.38
Eructation	6	3374	1.02	2.10 (0.94)	0.0019	2.89	12.57	4.44
Myocardial Ischaemia	5	3374	1.76	1.28 (0.02)	0.0011	23.29	2.84	2.07
Aspiration	5	3374	0.95	1.92 (0.65)	0.0012	4.85	5.24	3.63
Oesophagitis	5	3374	0.70	2.19 (0.93)	0.0014	8.75	8.53	4.53
Ageusia	5	3374	1.64	1.36 (0.10)	0.0013	12.62	3.05	2.23
Hypocalcaemia	4	3374	1.18	1.42 (0.01)	0.0010	2.50	4.19	2.13
Hypertensive Crisis	4	3374	0.76	1.84 (0.43)	0.0011	6.50	6.82	3.16
Hyperammonaemia	3	3374	0.48	1.84 (0.21)	0.0008	5.29	6.29	2.93

ADE, adverse drug events; PT, preferred term; *n*_111_, the number of reports of the specific ADE where both drug classes were used concomitantly; *n*_11+_, the total number of adverse-event records across all preferred terms where both drug classes were used concomitantly; *E*_111_, the expected value in the Ω shrinkage measure model; Ω, Ω shrinkage measure model; Ω_025_ > 0 is used as a threshold to screen for DDI signals under the combination of two drugs; AM, additive model; MM, multiplicative model; CRR, combination risk ratio model; χ, chi-square statistic derived from the combination model; AM is reported to four decimal places and the other measures to two decimal places.

**Table 3:** Parameters for calculating the probability density function of time-to-onset based on the Weibull distribution.

Number			Weibull Distribution				Failure Type
	TTO (Days)		Shape Parameter		Scale Parameter		
	Median (IQR)	Min–Max	α	95% CI	β	95% CI	
778	4 (0–21)	0–3653	0.52	0.49–0.55	34.12	28.25–39.98	Early failure

TTO, Time-to-onset.

**Table 4:** Logistic regression models of IMEs.

Variable	Logistics Regression							
	Univariate				Multivariate ^†^			
	OR	95% CI	*P*	AIC	OR	95% CI	*P*	AIC
Age (Continuous variables)	1.01	1.00, 1.02	0.013 *	1314	1.04	1.02, 1.06	<0.001 *	714
Age (Reference 18–60 years)								
≥60 years	1.34	1.04, 1.73	0.026 *	1316	2.45	1.72, 3.49	<0.001 *	715
Weight (Continuous variables)	1.00	0.99, 1.01	0.9	738	1.00	0.99, 1.01	0.8	714
Weight (Reference <67 kg)								
≥67 kg	0.98	0.69, 1.38	>0.900	738	0.96	0.68, 1.37	0.8	714
Gender (Reference Female)								
Male	1.10	0.85, 1.43	0.5	1318	0.94	0.65, 1.36	0.7	714

IME, important medical event; CI, Confidence Interval; OR, Odds Ratio; AIC, Akaike Information Criterion. All models were fitted to the 965-report analysis dataset using the R glm function (Stats package, R, version 4.5.1); missing values were handled by the function’s default complete-case procedure (na.action = na.omit). † Another variable in addition to the independent variables are used as covariates to adjust. When age and weight are used as covariates, we include continuous variables. * *P* < 0.05.

**Table 5:** The 4 × 2 contingency table for signal detection and calculation formula and threshold.

	Target ADE	Other ADEs	Total
Concomitant use of DrugA (5-HT3 RAs) and DrugB (NK-1 RAs)	*n*_111_	*n*_110_	*n*_11+_
DrugA (5-HT3 RAs) without DrugB (NK-1 RAs)	*n*_101_	*n*_100_	*n*_10+_
DrugB (NK-1 RAs) without DrugA (5-HT3 RAs)	*n*_011_	*n*_010_	*n*_01+_
Neither DrugA (5-HT3 RAs) or DrugB (NK-1 RAs)	*n*_001_	*n*_000_	*n*_00+_
Total	*n*_++1_	*n*_++0_	*n*_+++_

ADE: adverse drug events; 5-HT3 RAs: 5-HT3 receptor antagonists; NK-1 RAs: NK-1 receptor antagonists.

## Data Availability

The datasets analyzed during the current study are available in the FAERS database repository (https://open.fda.gov/data/faers/, accessed on 20 May 2025).

## Supplementary Materials

The following supporting information can be downloaded at https://www.mdpi.com/article/10.3390/ph19101626/s1. Table S1: Number of indication reports; Table S2: Number of reports on combined drug use; Table S3: Results of drug–drug interaction signal analysis of ADE signals; Table S4: Results of drug–drug interaction signal analysis of significant ADE signals (including primary suspected and second suspected cases); Table S5: Detailed drug terms utilized in the search through the FAERS database; Table S6: Probability density formulas based on the Weibull distribution.

## Abbreviations

The following abbreviations are used in this manuscript:

CINV	Chemotherapy-induced nausea and vomiting
5-HT3 RAs	5-Hydroxytryptamine Type 3 Receptor Antagonists
NK-1 RAs	Neurokinin-1 Receptor Antagonists
DDI	Drug–drug Interactions
ADEs	Adverse drug events
FDA	Food and Drug Administration
FAERS	Food and Drug Administration Adverse Event Reporting System
TTO	Time to onset
PT	Preferred term
IME	Important medical events
MASCC	Multinational Association of Supportive Care in Cancer
ESMO	European Society of Medical Oncology
ASCO	American Society of Clinical Oncology
NCCN	National Comprehensive Cancer Network
MEC	Moderately emetogenic chemotherapy
HEC	Highly emetogenic chemotherapy
PS	Primary suspected
SS	Second suspected
C	Concomitant
I	Interacting
MedDRA	Medical Dictionary for Regulatory Activities
SP	Substance P
